# Association of constipation with increased risk of hypertension and cardiovascular events in elderly Australian patients

**DOI:** 10.1038/s41598-023-38068-y

**Published:** 2023-07-06

**Authors:** Courtney P. Judkins, Yutang Wang, Maria Jelinic, Alex Bobik, Antony Vinh, Christopher G. Sobey, Grant R. Drummond

**Affiliations:** 1 Centre for Cardiovascular Biology and Disease Research and Department of Microbiology, Anatomy, Physiology & Pharmacology, School of Agriculture, Biomedicine & Environment, La Trobe University, Melbourne, VIC 3086 Australia; 2 Discipline of Life Science, Institute of Innovation, Science and Sustainability, Federation University Australia, Ballarat, VIC 3350 Australia; 3 Baker Heart and Diabetes Institute, Melbourne, VIC 3004 Australia

**Keywords:** Cardiovascular biology, Hypertension, Vascular diseases

## Abstract

The association between constipation and cardiovascular risk is unclear. This population-level matched cohort study compared the association of constipation with hypertension and incident cardiovascular events in 541,172 hospitalized patients aged ≥ 60 years. For each constipation admission, one exact age-matched non-constipated admission was randomly selected from all hospitalizations within 2 weeks to form the comparison cohort. The association of constipation with hypertension and cardiovascular events (myocardial infarction, angina, stroke and transient ischemic attack) were analysed using a series of binary logistic regressions adjusting for age, sex, cardiovascular risk factors, gastrointestinal disorders and sociological factors. Patients with constipation had a higher multivariate-adjusted risk for hypertension (odds ratio [OR], 1.96; 95% confidence interval [CI] 1.94–1.99; *P* < 0.001). Compared to patients with neither constipation nor hypertension, there was a higher multivariate-adjusted risk for cardiovascular events in patients with constipation alone (OR, 1.58; 95% CI 1.55–1.61; *P* < 0.001) or hypertension alone (OR, 6.12; 95% CI 5.99–6.26; *P* < 0.001). In patients with both constipation and hypertension, the risk for all cardiovascular events appeared to be additive (OR, 6.53; 95% CI 6.40–6.66; *P* < 0.001). In conclusion, among hospital patients aged 60 years or older, constipation is linked to an increased risk of hypertension and cardiovascular events. These findings suggest that interventions to address constipation may reduce cardiovascular risk in elderly patients.

## Introduction

The number of people with cardiovascular diseases (CVD) has nearly doubled over the past 30 years, and the number of deaths from CVD has increased from 12.1 million to 18.6 million during this time^1^. Despite efforts to modify traditional risk factors for CVD with lifestyle and drug interventions, cardiovascular events are still responsible for 32% of global deaths, 85% of which are due to myocardial infarction or stroke^2^. Therefore, identifying non-traditional CVD risk factors and developing strategies to address them is critical to further reduce CVD-associated morbidity and mortality^3^.

Hypertension is the most important modifiable risk factor for cardiovascular events^4^ and its prevalence in the elderly is much higher than in younger people^5^. Another common condition in the elderly is constipation^6^. Although it is unclear whether constipation is independently associated with hypertension, a relationship is plausible because in constipation there is increased water absorption from the gut^7^, microbiota changes^8^, and inflammation^9–11^, all of which could lead to hypertension. It also remains unclear whether constipation is an independent risk factor for cardiovascular events with conflicting reports appearing in the literature. For example, the Ohsaki Cohort study^12^ reported that constipation was associated with a higher risk for stroke mortality and CVD mortality overall in Japanese people. Similarly, Sumida et al^13^ found that constipation was associated with a higher incidence of coronary heart disease and ischemic stroke in US veterans. By contrast, the US Nurses’ Health Study reported no significant association between infrequent bowel movements (i.e. every 3 days or less) and cardiovascular disease^14^. While the reasons for these discrepancies remain unclear, it is noteworthy that the latter study only included female participants^14^.

The current study aimed to investigate whether constipation is associated with hypertension and cardiovascular events in an Australian cohort comprising 541,172 hospital patients of either sex and aged ≥ 60 years – an age distribution in which cardiovascular events are most prevalent. We also examined whether the presence of hypertension modifies the association between constipation and cardiovascular events.

## Results

### General characteristics

This study included 541,172 hospital patients, half of whom had constipation and of which 45.4% were males (Table 1). The mean (SD) age was 73.7 (8.4) years. 49.1% of patients with constipation also had hypertension (Table 1), and 68.1% of patients with hypertension also had constipation (Supplementary Table 1). Compared with those without constipation, patients with constipation were more likely to have hypertension and other CVD comorbidities including obesity, smoking, diabetes, sleep apnoea, atrial fibrillation and arrhythmia (AFA), peripheral occlusive arterial disease (POAD), chronic obstructive pulmonary disease (COPD), kidney disease, endocrine and metabolic disorders (Table 1). The percentage of patients with constipation who had one or more cardiovascular events, including myocardial infarction, angina, stroke and/or transient ischemic attack, was higher than that of patients without constipation (Table 1).

**Table 1:** Characteristics of patients, stratified by constipation.

	All patients	Patients with constipation	Patients without constipation	*P* value
Sample size	541,172	270,586	270,586	NA
Male, n (%)	245,650 (45.4)	122,825 (45.4)	122,825 (45.4)	1.000
Age, mean (SD)	73.7 (8.4)	73.7 (8.4)	73.7 (8.4)	1.000
All cardiovascular events,^a^ n (%)	172,634 (31.9)	112,649 (41.6)	59,985 (22.2)	< 0.001
Myocardial infarction, n (%)	115,863 (21.4)	75,806 (28.0)	40,057 (14.8)	< 0.001
Angina, n (%)	37,262 (6.9)	26,404 (9.8)	10,858 (4.0)	< 0.001
Stroke, n (%)	62,288 (11.5)	42,715 (15.8)	19,573 (7.2)	< 0.001
TIA, n (%)	21,868 (4.0)	15,509 (5.7)	6,359 (2.3)	< 0.001
Hypertension, n (%)	195,062 (36.0)	132,889 (49.1)	62,173 (23.0)	< 0.001
Obesity, n (%)	28,400 (5.2)	20,001 (7.4)	8,399 (3.1)	< 0.001
Smoking, n (%)	235,013 (43.4)	139,600 (51.6)	95,413 (35.3)	< 0.001
Diabetes, n (%)	123,103 (22.7)	75,619 (27.9)	47,484 (17.5)	< 0.001
Sleep apnoea, n (%)	18,730 (3.5)	12,602 (4.7)	6,128 (2.3)	< 0.001
AFA, n (%)	135,691 (25.1)	91,790 (33.9)	43,901 (16.2)	< 0.001
POAD, n (%)	25,718 (4.7)	18,536 (6.8)	7,182 (2.6)	< 0.001
COPD, n (%)	34,280 (6.3)	26,381 (9.7)	7,899 (2.9)	< 0.001
Kidney disease, n (%)	152,966 (28.3)	111,341 (41.1)	41,625 (15.4)	< 0.001
Endocrine disorders, n (%)	165 (0.03)	118 (0.04)	47 (0.02)	< 0.001
Metabolic disorders, n (%)	15,377 (2.8)	12,049 (4.4)	3,328 (1.2)	< 0.001
Irritable bowel syndrome	2,747 (0.51)	1,993 (0.74)	754 (0.28)	< 0.001
Ulcerative colitis	3,509 (0.65)	2,259 (0.83)	1,250 (0.46)	< 0.001
Crohn’s disease	1,732 (0.32)	1,151 (0.43)	581 (0.21)	< 0.001
Other gastrointestinal disorders	116,578 (21.5)	80,039 (29.6)	36,539 (13.5)	< 0.001
Metropolitan residence, n (%)	310,621 (67.6)	163,729 (68.7)	146,892 (66.4)	< 0.001

AFA, atrial fibrillation and arrhythmia; COPD, chronic obstructive pulmonary disease; CP, constipation; CVD, cardiovascular disease; POAD, peripheral occlusive arterial disease; TIA, transient ischemic attack.

^a^All cardiovascular events included myocardial infarction, angina, stroke and transient ischemic attack.

When the data were stratified by hypertension, the proportions of patients with hypertension who had constipation, cardiovascular events and comorbidities (obesity, smoking, diabetes, sleep apnoea, AFA, POAD, COPD, kidney disease, endocrine and metabolic disorders) were higher than in patients without hypertension (Supplementary Table 1).

### Association of constipation with hypertension and cardiovascular events after adjustment for biological and sociological factors

Compared to those without constipation, patients with constipation had a higher risk for hypertension (OR, 1.96; 95% CI 1.94–1.99; *P* < 0.001; Table 2) after adjustment for age, sex, cardiovascular risk factors (CV risk factors; obesity, smoking, diabetes, sleep apnoea, COPD, kidney disease, endocrine and metabolic disorders, POAD, AFA), gastrointestinal disorders (GI disorders; irritable bowel syndrome, ulcerative colitis, Crohn’s disease and other gastrointestinal disorders) and metropolitan residence. Across all participants, constipation was also associated with a higher risk for all cardiovascular events (OR, 1.32; 95% CI 1.30–1.34; *P* < 0.001; Table 3), myocardial infarction (OR, 1.10; 95% CI 1.09–1.12; *P* < 0.001; Table 3) and stroke (OR, 1.51; 95% CI 1.48–1.54; *P* < 0.001; Table 3) after adjustment for age, sex, CV risk factors, GI disorders and metropolitan residence.

**Table 2:** Association of constipation (predictor variable) with hypertension (outcome variable).

	OR	95% CI	*P* value
Model 1	3.23	3.20–3.27	< 0.001
Model 2	3.23	3.20–3.27	< 0.001
Model 3	2.04	2.01–2.06	< 0.001
Model 4	1.97	1.94–1.99	< 0.001
Model 5	1.96	1.94–1.99	< 0.001

CI, confidence interval; COPD, chronic obstructive pulmonary disease; OR, odds ratio; POAD, peripheral occlusive arterial disease.

Model 1: Adjusted for age.

Model 2: Adjusted for age and sex.

Model 3: Adjusted for age, sex, and cardiovascular risk factors (obesity, smoking, diabetes, sleep apnoea, COPD, kidney disease, endocrine disorders, metabolic disorders, POAD, atrial fibrillation and cardiac arrhythmia).

Model 4: Adjusted for all the factors in Model 3 plus gastrointestinal disorders (irritable bowel syndrome, ulcerative colitis, Crohn’s disease and other gastrointestinal disorders).

Model 5: Adjusted for all the factors in Model 4 plus metropolitan residence.

**Table 3:** Risk of major cardiovascular events associated with constipation in all 541,172 participants.

	OR	95% CI	*P* value^a^
All cardiovascular events^b^			
Model 1	2.50	2.48–2.53	< 0.001
Model 2	2.52	2.49–2.55	< 0.001
Model 3	1.34	1.32–1.36	< 0.001
Model 4	1.32	1.30–1.34	< 0.001
Model 5	1.32	1.30–1.34	< 0.001
Myocardial infarction			
Model 1	2.24	2.21–2.27	< 0.001
Model 2	2.26	2.23–2.29	< 0.001
Model 3	1.12	1.10–1.14	< 0.001
Model 4	1.11	1.09–1.12	< 0.001
Model 5	1.10	1.09–1.12	< 0.001
Stroke			
Model 1	2.41	2.36–2.45	< 0.001
Model 2	2.41	2.36–2.45	< 0.001
Model 3	1.51	1.48–1.54	< 0.001
Model 4	1.51	1.48–1.54	< 0.001
Model 5	1.51	1.48–1.54	< 0.001

CI, confidence interval; COPD, chronic obstructive pulmonary disease; HT, hypertension; OR: odds ratio; POAD, peripheral occlusive arterial disease.

^a^The significance of OR for major cardiovascular events associated with constipation.

^b^Cardiovascular events included myocardial infarction, angina, stroke and transient ischemic attack.

Model 1: Adjusted for age.

Model 2: Adjusted for age and sex.

Model 3: Adjusted for age, sex, and cardiovascular risk factors (hypertension, obesity, smoking, diabetes, sleep apnoea, COPD, kidney disease, endocrine disorders, metabolic disorders, POAD, atrial fibrillation and cardiac arrhythmia).

Model 4: Adjusted for all the factors in Model 3 plus gastrointestinal disorders (irritable bowel syndrome, ulcerative colitis, Crohn’s disease and other gastrointestinal disorders).

Model 5: Adjusted for all the factors in Model 4 plus metropolitan residence.

### Hypertension modified the association of constipation with cardiovascular events

**Figure 1:**
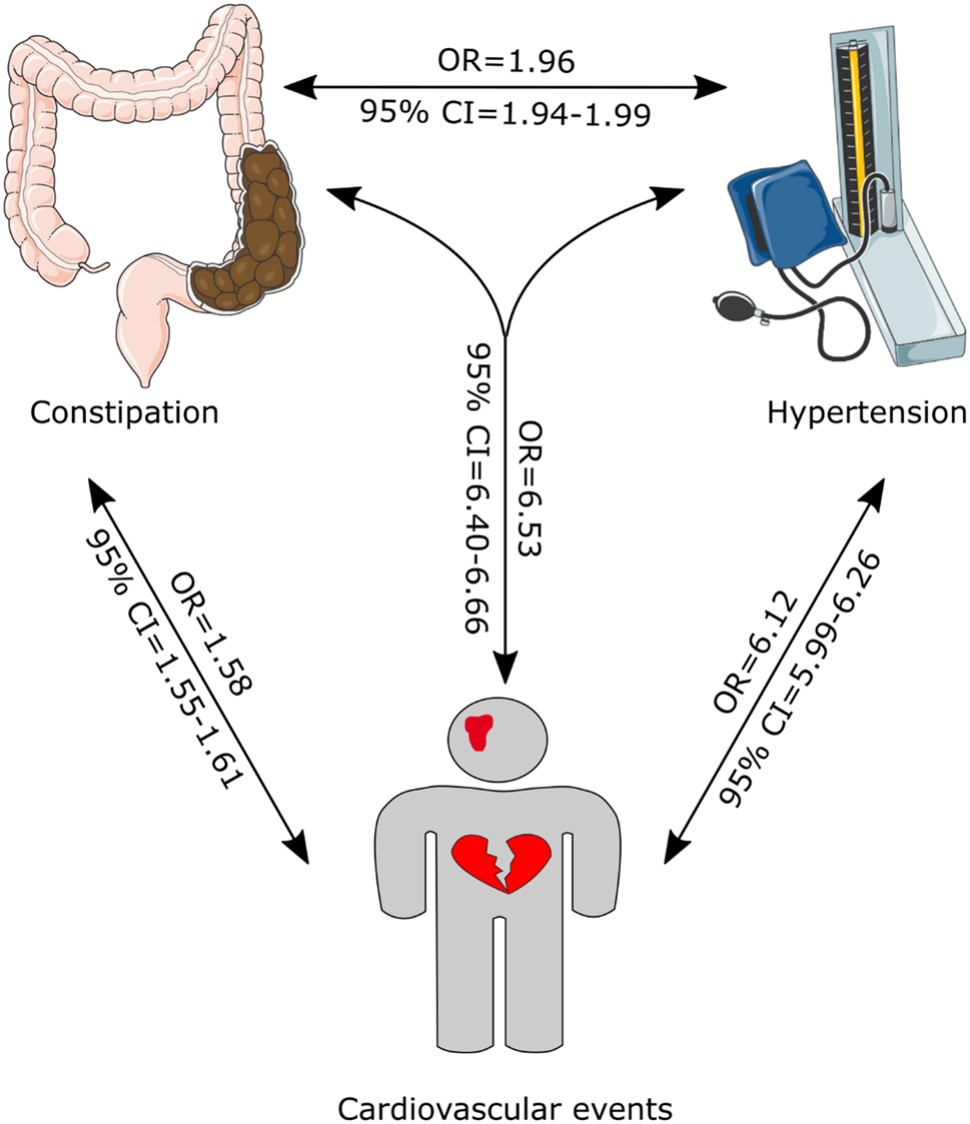
Summary of the findings. This study found that constipation was positively associated with hypertension and cardiovascular events. CI, confidence interval; OR, odds ratio. This figure was partly generated using Servier Medical Art, provided by Servier, licensed under a Creative Commons Attribution 3.0 unported license.

Binary logistical regression analysis indicated that there was an interaction between hypertension and constipation in modulating the risk of all cardiovascular events, myocardial infarction and stroke (Supplementary Table 2). Therefore, we evaluated the individual and combined associations of constipation and/or hypertension with cardiovascular events (Table 4). Compared to patients with neither constipation nor hypertension, patients with constipation alone had a higher multivariate-adjusted risk for cardiovascular events (OR, 1.58; 95% CI 1.55–1.61; *P* < 0.001; Table 4), myocardial infarction (OR, 1.35; 95% CI 1.32–1.38; *P* < 0.001; Table 4) and stroke (OR, 2.00; 95% CI 1.94–2.06; *P* < 0.001; Table 4). Patients with hypertension alone also had a higher multivariate-adjusted risk for cardiovascular events (OR, 6.12; 95% CI 5.99–6.26; *P* < 0.001; Table 4), myocardial infarction (OR, 4.83; 95% CI 4.72–4.95; *P* < 0.001; Table 4) and stroke (OR, 5.38; 95% CI 5.22–5.56; *P* < 0.001; Table 4). In patients with both constipation and hypertension, the risk for all cardiovascular events appeared to be additive (OR, 6.53; 95% CI 6.40–6.66; *P* < 0.001; Table 4**)**, as did that for stroke (OR, 6.82; 95% CI 6.62–7.02; *P* < 0.001; Table 4 and Fig. 1). These risk profiles were similar in males and females (Supplementary Tables 3 and 4).

**Table 4:** Risk of cardiovascular events associated with constipation and hypertension in all 541,172 patients.

	Without either condition (N = 208,413)	With constipation alone (N = 137,697)			With hypertension alone (N = 62,173)			With both constipation and hypertension (N = 132,889)		
	OR	OR	95% CI	*P* value^a^	OR	95% CI	*P* value^a^	OR	95% CI	*P* value^a^
All cardiovascular events^b^										
Model 1	1.00 (reference)	2.07	2.03–2.11	< 0.001	8.37	8.20–8.54	< 0.001	10.99	10.80–11.18	< 0.001
Model 2	1.00 (reference)	2.07	2.03–2.11	< 0.001	8.54	8.37–8.72	< 0.001	11.34	11.15–11.54	< 0.001
Model 3	1.00 (reference)	1.61	1.58–1.64	< 0.001	6.23	6.10–6.37	< 0.001	6.71	6.58–6.84	< 0.001
Model 4	1.00 (reference)	1.59	1.56–1.62	< 0.001	6.19	6.06–6.33	< 0.001	6.59	6.46–6.72	< 0.001
Model 5	1.00 (reference)	1.58	1.55–1.61	< 0.001	6.12	5.99–6.26	< 0.001	6.53	6.40–6.66	< 0.001
Myocardial infarction										
Model 1	1.00 (reference)	1.87	1.83–1.91	< 0.001	7.28	7.12–7.45	< 0.001	8.81	8.64–8.98	< 0.001
Model 2	1.00 (reference)	1.87	1.83–1.91	< 0.001	7.44	7.27–7.61	< 0.001	9.13	8.95–9.31	< 0.001
Model 3	1.00 (reference)	1.37	1.34–1.41	< 0.001	4.93	4.81–5.05	< 0.001	4.68	4.58–4.79	< 0.001
Model 4	1.00 (reference)	1.35	1.32–1.38	< 0.001	4.89	4.77–5.01	< 0.001	4.58	4.48–4.68	< 0.001
Model 5	1.00 (reference)	1.35	1.32–1.38	< 0.001	4.83	4.72–4.95	< 0.001	4.54	4.44–4.64	< 0.001
Stroke										
Model 1	1.00 (reference)	2.20	2.13–2.27	< 0.001	6.23	6.04–6.42	< 0.001	8.41	8.20–8.63	< 0.001
Model 2	1.00 (reference)	2.19	2.13–2.26	< 0.001	6.23	6.04–6.42	< 0.001	8.44	8.23–8.67	< 0.001
Model 3	1.00 (reference)	2.00	1.94–2.07	< 0.001	5.42	5.25–5.59	< 0.001	6.85	6.66–7.05	< 0.001
Model 4	1.00 (reference)	2.00	1.94–2.07	< 0.001	5.42	5.25–5.59	< 0.001	6.85	6.66–7.06	< 0.001
Model 5	1.00 (reference)	2.00	1.94–2.06	< 0.001	5.38	5.22–5.56	< 0.001	6.82	6.62–7.02	< 0.001

CI, confidence interval; COPD, chronic obstructive pulmonary disease; HT, hypertension; OR: odds ratio; POAD, peripheral occlusive arterial disease.

^a^The significance of OR for major cardiovascular events associated with constipation.

^b^Cardiovascular events included myocardial infarction, angina, stroke and transient ischemic attack.

Model 1: Adjusted for age.

Model 2: Adjusted for age and sex.

Model 3: Adjusted for age, sex, and cardiovascular risk factors (obesity, smoking, diabetes, sleep apnoea, COPD, kidney disease, endocrine disorders, metabolic disorders, POAD, atrial fibrillation and cardiac arrhythmia).

Model 4: Adjusted for all the factors in Model 3 plus gastrointestinal disorders (irritable bowel syndrome, ulcerative colitis, Crohn’s disease and other gastrointestinal disorders).

Model 5: Adjusted for all the factors in Model 4 plus metropolitan residence.

To validate the above findings, sensitivity analyses were performed in which only those patients coded with primary hypertension were included, as opposed to including patients with all forms of hypertension. Using this variable did not materially change the multivariate-adjusted association of constipation with hypertension (Supplementary Table 5) or the independent and combined risk profiles of hypertension and constipation with all cardiovascular events (Supplementary Table 6).

## Discussion

In an Australian cohort comprising 541,172 hospital patients aged ≥ 60 years, we found that constipation was positively and independently associated with hypertension, and that the two factors interacted to modify the risk of cardiovascular events. Thus, patients with either constipation or hypertension alone had an increased risk of myocardial infarction, stroke and all cardiovascular events, compared to patients with neither condition. Patients with both constipation and hypertension had an even higher risk of cardiovascular events. These relationships were similar in males and females.

Constipation is one of the most frequently reported gastrointestinal disorders and has a major bearing on measures of health-related quality of life. Estimates of the prevalence of constipation in the population range between 2 and 26%, depending on the definition and diagnostic approach used to evaluate the condition. Furthermore, there is substantial evidence that its prevalence increases with age^15^. In our study, among 1,783,246 patients over 60 years, the prevalence of constipation, as defined by hospital coding data (ICD-10 K59.0), was 15.2%.

There are a number of plausible mechanistic links between constipation and hypertension (see below). However, besides an earlier study in which constipation was identified as an independent predictor of hypertension in children occurring as a result of therapy for acute lymphoblastic leukaemia^16^, to our knowledge this is one of the first studies to report an association between the two conditions. There are several potential mechanisms that might explain this positive association. For example, increased water absorption from the gut during constipation could increase blood volume which may in turn lead to hypertension^7^. Constipation can also promote gut dysbiosis, which has emerged as a vital modulator of many pathophysiological processes implicated in the pathogenesis of hypertension including inflammation and altered production of vasoactive short-chain fatty acids^9–11^. Studies dating back to the 1960s reported that complete obstruction of the gut promotes bacterial overgrowth above the obstruction, while a normal intestinal flora exists below it^17^. These findings were recapitulated in animal models of intestinal obstruction where 16 s RNA sequencing of the gut microbiota revealed striking alterations in bacterial community diversity and richness^18^. Of course, we cannot exclude the alternative possibility that hypertension, or its therapeutic management, directly or indirectly caused constipation. Indeed, hypertension is frequently associated with sympathetic hyperactivity^19^, which might be expected to reduce colonic motility. Moreover, constipation is an adverse effect of some anti-hypertensive medications such as calcium channel blockers^20^ and diuretics^21^. Hence, further investigations with preclinical models and prospective human trials will be necessary to determine if causality and directionality exist between hypertension and constipation. For example, it would be interesting to test if laxative use in hypertensive patients with constipation reduces blood pressure and cardiovascular events, but to our knowledge no such study has been performed.

We also found that constipation was positively associated with myocardial infarction, stroke and all cardiovascular events, which is consistent with earlier reports^12,13^. While we can only speculate on whether a mechanistic link exists between constipation and cardiovascular events, there are a number of potential explanations that might connect the two. As discussed, constipation can promote gut dysbiosis^22^, which is recognised as a contributor to systemic inflammation and cardiovascular risk^23–25^. In addition, patients with constipation can experience strain at stool, and this has been shown to increase blood pressure acutely by up to 70 mm Hg^26^. Such acute pressor responses could conceivably trigger a cardiovascular event. Constipation was also associated with a higher prevalence of chronic hypertension and thus it is possible that the latter condition represents a mediator of cardiovascular events in patients with constipation. Indeed, we found that hypertension interacted with constipation in modifying the risk of cardiovascular events.

A previous study found no link between constipation and cardiovascular events^14^. However, it is noteworthy that this study included a large proportion of young female participants, with a median age of 48 years, and 45% of whom were pre-menopausal^14^. Similarly, the Japan Collaborative Cohort Study for Evaluation of Cancer (JACC) also found no association between constipation and cardiovascular disease in women, although it did find a link in Japanese men^27^. Again, a large proportion of the women in the JACC study were pre-menopausal^27^. In contrast, our study comprised people of both sexes over the age of 60. Two earlier studies, one that included mostly older women with a median age of 59^12^, and another that only included post-menopausal women^28^, also reported a link between constipation and cardiovascular events. Together, these findings suggest that in women, the link between constipation and cardiovascular events may become apparent after menopause.

### Strengths and limitations

The strengths of this study include its large sample size and a substantial period of observation in elderly people when constipation and cardiovascular events are more prevalent. However, given that the risk for stroke and myocardial infarction is heavily influenced by age, we matched 1:1 on age to remove its confounding influence. We additionally controlled for the influence of other vascular and gastrointestinal factors and attempted to partition their influence on cerebrovascular and coronary event risk. We used well-audited and standard methods of diagnostic coding to identify constipation and outcomes, with established data linkage procedures.

Limitations of the study could include the possibility of misclassification of diseases in the database. However, for each hospital admission, the coding was performed according to the International Classification of Diseases, 10th Revision after the patient’s episode of care by experienced hospital coders. The quality of the hospital data was maintained using an independent audit program and a previous study has shown that the coding quality for diagnosis in Victorian public hospital data had very high accuracy; for example, kappa was 0.91 between coding auditors and hospital coders for the diagnosis of cardiovascular events^29^. Regardless of the quality of coding, such hospital data do not provide any information on parameters such as duration nor severity of any of these conditions. Another limitation is that the study used hospitalised patients, and therefore, the findings may not apply to the general population. Also, as alluded to above, certain anti-hypertensive medications can cause constipation, and it is conceivable that this may contribute to the association between hypertension and constipation. Similarly, laxative use has been linked with cardiovascular events. However, the database used for this study was not linked to records of prescribed drug use, and thus we are unable to evaluate the influence of medication on the current findings. It is also noteworthy that the large sample size could tend to contribute to statistical significance being achieved across coefficients, but this does not necessarily equate to clinical significance. Finally, the cohort of this study was, by its nature, highly heterogeneous. To control for this, we adjusted for multiple cardiovascular, gastrointestinal and socioeconomic factors. We also separated primary hypertensives from the total hypertensive group, and showed that the associations with constipation still held for this group.

In conclusion, among hospital patients aged 60 years or older, constipation is linked to an increased risk for hypertension, stroke, myocardial infarction, and all cardiovascular events. Compared to patients with neither constipation nor hypertension, there was a higher risk for cardiovascular events in patients with either constipation or hypertension alone, and an additive risk in patients with both conditions. Further research is necessary to establish whether there is a direct causal relationship between constipation and cardiovascular disease. If such a connection is confirmed, it may be necessary to consider dietary or therapeutic interventions (e.g. laxatives) aimed at relieving constipation as part of the clinical management strategy for reducing cardiovascular risk in elderly patients.

## Methods

### Study participants

**Figure 2:**
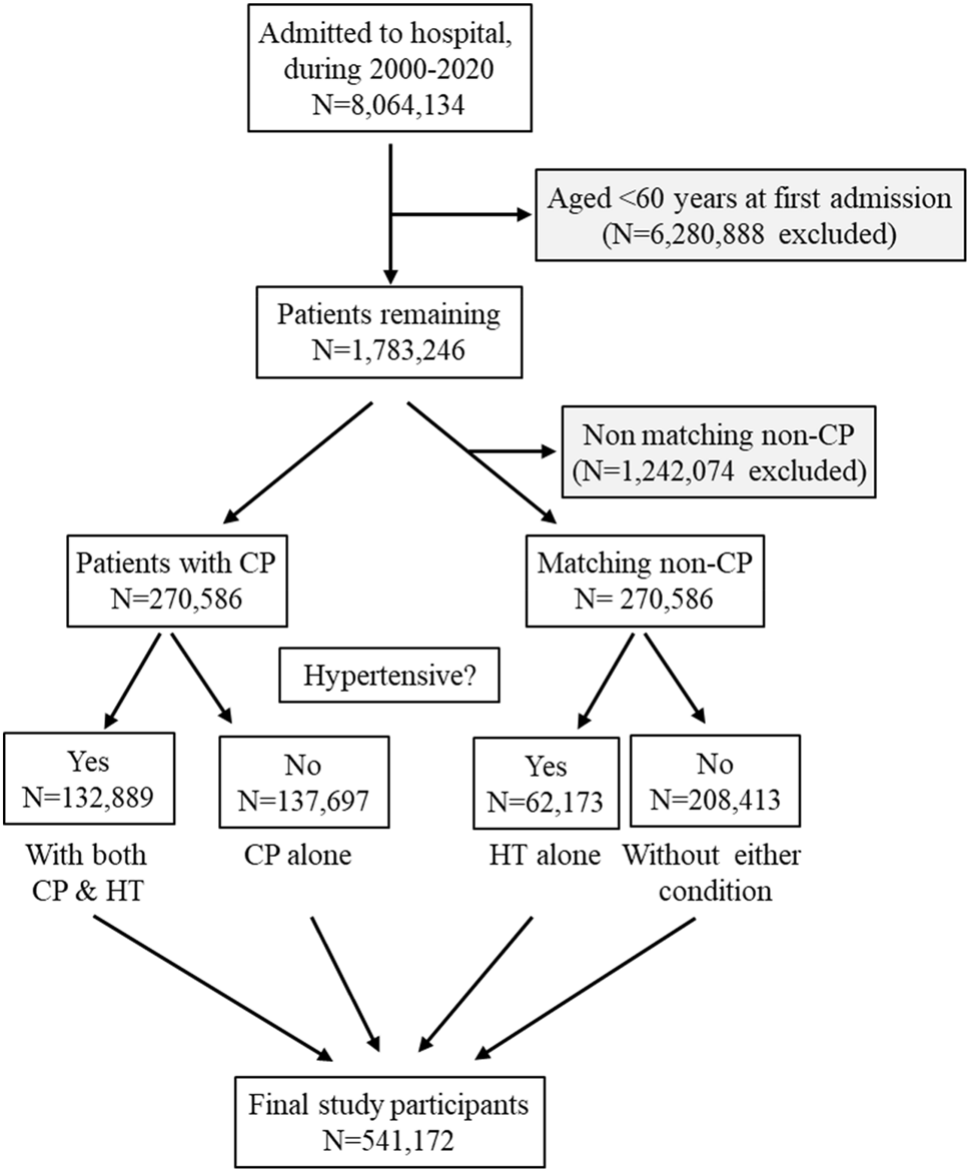
Flow diagram of the study participants. CP, constipation; HT, hypertension.

The data in this study were obtained from the Data Custodian of the Centre for Victorian Data Linkage, the Department of Health and Human Services of Victoria^30^. The data were derived from all public and private hospitalizations in Victoria, a state of Australia with a population of > 6 million (approx. 25% of the Australian population). Australians have universal access to hospital medical care and the reporting of disease diagnosis is mandatory^30^. From 1 July 2000 to 30 June 2020, 8,064,1234 patients were recorded to be admitted to Victorian public and private hospitals in the dataset, from which 6,280,888 were excluded on the basis of being aged < 60 years at first admission (Fig. 2). Among the remaining 1,783,246 patients, 270,586 patients had constipation. A total of another 270,586 who did not have constipation but were of the same age and sex were chosen as matching non-constipated patients. Therefore, this analysis included 541,172 patients aged ≥ 60 years in the final analysis, half of whom had constipation (Fig. 2).

### Ethical considerations

This study was approved by La Trobe University Human Research Ethics Committee (approval number, HEC20380). All procedures were performed following the guidelines of the Declaration of Helsinki. Patient information was anonymized and de-identified prior to analysis and written informed consent was waived by the La Trobe University Human Research Ethics Committee.

### Constipation

Constipation (K59.0) was identified according to the International Classification of Diseases, 10th Revision (Supplementary Table 7).

### Cardiovascular events

Cardiovascular events in the current study included myocardial infarction, angina, stroke, and transient ischemic attack. Myocardial infarction (I21, I22, I23, I24, I25), angina (I200), stroke (G46, I60, I61, I62, I63, I64, I65, I66, I67), and transient ischaemic attack (G450-453, G458-459) were diagnosed according to the International Classification of Diseases, 10th Revision (Supplementary Table 7).

### Covariates

Confounding covariates included age (continuous), sex (male or female), obesity (yes or no, defined as BMI ≥ 30), smoking (yes or no), hypertension (yes or no), diabetes (yes or no), sleep apnoea (yes or no), COPD (yes or no), kidney disease (yes or no), endocrine disorder (yes or no), metabolic disorder (yes or no), POAD (yes or no), atrial fibrillation and cardiac arrhythmia (yes or no), irritable bowel syndrome (yes or no), ulcerative colitis (yes or no), Crohn’s disease (yes or no), other gastrointestinal diseases (yes or no) and metropolitan residence (yes or no) which is a proxy for socioeconomic status^31^. Obesity (E66, U781), smoking (Z716, Z720, Z812, Z8643, T652), hypertension (I10, I11, I12, I13, I15), diabetes (E10, E11, E13, E14), sleep apnoea (G473, G474, G478, G479), COPD (U832), kidney disease (I12, I13, I150, I151, N17, N18, N19, Q61, U871), endocrine disorder (E34, G735, I152), metabolic disorders (E88, E89, G736, I431, M141, N163, U782), POAD (I702), atrial fibrillation and cardiac arrhythmia (I48, I49) irritable bowel syndrome (K58), ulcerative colitis (K51,U842), Crohn’s disease (K50, U841), other gastrointestinal diseases (A213, A222, B462, K63, K9, M9836, P543, Q6475, T478, T479) were diagnosed according to the International Classification of Diseases, 10th Revision^32^ (Supplementary Table 7). These covariates were associated with cardiovascular events analysed by simple binary logistic regression (Supplementary Table 8).

### Statistical analyses

Data were presented as mean and standard deviation for continuous variables, or number and percentage for categorical variables^33^. Differences in age between two groups were analysed using Student’s t-test. Differences among categorical variables were analysed using Fisher’s exact test^34,35^. We tested the hypothesis that constipation was associated with hypertension and cardiovascular events using binary logistic models^36^ controlling for age, sex, obesity, smoke, diabetes, sleep apnoea, COPD, kidney disease, endocrine and metabolic disorders, POAD, atrial fibrillation and arrhythmia, irritable bowel syndrome, ulcerative colitis, Crohn’s disease, other gastrointestinal disorders and metropolitan residence. Sub-analyses were conducted in sub-groups of patients stratified by hypertension or sex. Interaction of hypertension with constipation in modulating the risk of cardiovascular events was analysed by binary logistic regression with cardiovascular events as the outcome variable. In the interaction analysis, a new variable (interaction factor) was computed as constipation multiplied by hypertension. Constipation, hypertension, and the computed interaction factor were adjusted simultaneously along with other co-variables in the logistic regression and a P value of < 0.05 for the interaction factor in the regression analysis established that there was an interaction between hypertension with constipation in modulating the risk of cardiovascular events^37^. The null hypothesis was rejected for two-sided *P* values of < 0.05^38^. All analyses were performed using Stata/SE 17 (Standard Edition 17.0, Stata Corporation, College Station, Texas, USA). This study had a large sample size and this feature may tend to show significance across the coefficients. Therefore, readers should perceive carefully statistical significance versus clinical significance.

## Supplementary Information

Supplementary Information.

## Data Availability

The datasets are available from the corresponding author upon approval from the Data Custodian of the Centre for Victorian Data Linkage.
