# Supplementary material for: Association of constipation with increased risk of hypertension and cardiovascular events in elderly Australian patients
Source: Sci Rep. 2023 Jul 6;13:10943. doi: 10.1038/s41598-023-38068-y (PMC10326061; doi:10.1038/s41598-023-38068-y)
Supplement: Supplementary file 1 — Supplementary Information. [file 41598_2023_38068_MOESM1_ESM.docx]

**SUPPLEMENTARY MATERIAL**

**Association of c****onstipation with increased risk of hypertension and cardiovascular events in elderly Australian patients**

**Authors**: Courtney P. Judkins^1,*^, Yutang Wang^2,*^, Maria Jelinic^1^, Alex Bobik^1,3^, Antony Vinh^1^, Christopher G. Sobey^1,#^, Grant R. Drummond^1, #^

^1^Centre for Cardiovascular Biology and Disease Research and Department of Microbiology, Anatomy, Physiology & Pharmacology, School of Agriculture, Biomedicine & Environment, La Trobe University, Melbourne, Victoria, 3086, Australia

^2^Discipline of Life Science, Institute of Innovation, Science and Sustainability, Federation University Australia, Ballarat, Victoria, 3350, Australia.

^3^Baker Heart and Diabetes Institute, Melbourne, Victoria, 3004, Australia

* These authors contributed equally

#Co-senior/corresponding authors

**Correspondence:**

Professor Grant R. Drummond

Centre for Cardiovascular Biology and Disease Research

School of Agriculture, Biomedicine and Environment

La Trobe University

[g.drummond@latrobe.edu.au](mailto:g.drummond@latrobe.edu.au)

Professor Christopher G. Sobey

Centre for Cardiovascular Biology and Disease Research

School of Agriculture, Biomedicine and Environment

La Trobe University

[c.sobey@latrobe.edu.au](mailto:c.sobey@latrobe.edu.au)

**Running title**: Constipation, hypertension, and cardiovascular events

**Supplementary Table 1:** Characteristics of patients, stratified by hypertension

|  | All patients | Patients  with HT | Patients  without HT | P value^a^ |
| --- | --- | --- | --- | --- |
| Sample size | 541,172 | 195,062 | 346,110 | NA |
| Male, n (%) | 245,650 (45.4) | 87,810 (35.7) | 157,840 (64.3) | <0.001 |
| Age, mean (SD) | 73.7 (8.4) | 73.8 (7.8) | 73.7 (8.8) | <0.001 |
| Constipation, n (%) | 270,586 (50%) | 132,889 (68.1) | 137,697 (39.8) | <0.001 |
| All cardiovascular events ^b^, n (%) | 172,634 (31.9) | 115,077 (59.0) | 57,557 (16.6) | <0.001 |
| Myocardial Infarction, n (%) | 115,863 (21.4) | 80,262 (41.2) | 35,237 (10.2) | <0.001 |
| Angina, n (%) | 37,262 (6.9) | 29,084 (14.9) | 8,178 (2.4) | <0.001 |
| Stroke, n (%) | 62,288 (11.5) | 44,012 (22.6) | 18,276 (5.3) | <0.001 |
| TIA, n (%) | 21,868 (4.0) | 14,530 (7.4) | 7,338 (2.1) | <0.001 |
| Obesity, n (%) | 28,400 (5.2) | 18,811 (9.6) | 9,589 (2.8) | <0.001 |
| Smoking, n (%) | 235,013 (43.4) | 102,919 (52.8) | 132,094 (38.2) | <0.001 |
| Diabetes, n (%) | 123,103 (22.7) | 80,539 (41.3) | 42,564 (12.3) | <0.001 |
| Sleep apnoea, n (%) | 18,730 (3.5) | 10,506 (5.4) | 8,224 (2.4) | <0.001 |
| AFA, n (%) | 135,691 (25.1) | 80,708 (41.4) | 54,983 (15.9) | <0.001 |
| POAD, n (%) | 25,718 (4.7) | 18,456 (9.5) | 7,262 (2.1) | <0.001 |
| COPD, n (%) | 34,280 (6.3) | 19,025 (9.7) | 15,255 (4.4) | <0.001 |
| Kidney Disease, n (%) | 152,966 (28.3) | 97,950 (50.2) | 55,016 (15.9) | <0.001 |
| Endocrine Disorders, n (%) | 165 (0.03) | 102 (0.05) | 63 (0.02) | <0.001 |
| Metabolic Disorders, n (%) | 15,377 (2.8) | 8,735 (4.5) | 6,642 (1.9) | <0.001 |
| Irritable bowel syndrome | 2,747 (0.51) | 1,412 (0.72) | 1,335 (0.39) | <0.001 |
| Ulcerative colitis | 3,509 (0.65) | 1,699 (0.87) | 1,810 (0.52) | <0.001 |
| Crohn’s disease | 1,732 (0.32) | 858 (0.44) | 874 (0.25) | <0.001 |
| Other gastrointestinal disorders | 116,578 (21.5) | 57,947 (29.7) | 58,631 (16.9) | <0.001 |
| Metropolitan residence, n (%) | 310,621 (67.6) | 116,872 (59.9) | 193,749 (56.0) | <0.001 |

Abbreviations: AFA, atrial fibrillation and arrhythmia; COPD, chronic obstructive pulmonary disease; HT, hypertension; NA, not applicable; POAD, peripheral occlusive arterial disease; TIA, transient ischemic attack.

^a^ Comparison between those with and without hypertension

^b^ Cardiovascular events include myocardial infarction, angina, stroke and transient ischemic attack

**Supplementary Table 2**: Interaction of hypertension with constipation in modulating the risk of cardiovascular events, analysed by binary logistical regression

|  | All cardiovascular events ^a^ | | | Myocardial infarction | | | Stroke | | |
| --- | --- | --- | --- | --- | --- | --- | --- | --- | --- |
|  | OR ^b^ | 95% CI | P value ^c^ | OR ^b^ | 95% CI | P value ^c^ | OR ^b^ | 95% CI | P value ^c^ |
| CP x HT ^d^ | 0.67 | 0.66-0.69 | <0.001 | 0.70 | 0.68-0.72 | <0.001 | 0.63 | 0.61-0.66 | <0.001 |

Abbreviations: CI, confidence interval; COPD, chronic obstructive pulmonary disease; OR, odds ratio; POAD, peripheral occlusive arterial disease.

^a^ Cardiovascular events included myocardial infarction, angina, stroke and transient ischemic attack.

^b^ Adjusted for age, sex, cardiovascular risk factors (obesity, smoking, diabetes, sleep apnoea, COPD, kidney disease, endocrine disorders, metabolic disorders, POAD, atrial fibrillation and cardiac arrhythmia), gastrointestinal disorders (irritable bowel syndrome, ulcerative colitis, Crohn’s disease, and other gastrointestinal disorders), and metropolitan residence.

^c^ The significance of OR for major cardiovascular events associated with the interaction variable (constipation x hypertension).

^d^ Interaction factor, calculated as constipation (CP) multiplied by hypertension (HT).

**Supplementary Table 3**: Risk of cardiovascular events associated with constipation and hypertension in 245,650 male patients

|  | Without either condition (N=94,380) | With constipation alone  (N=63,460) | | | With hypertension alone  (N=28,445) | | | With both constipation and hypertension (N=59,365) | | |
| --- | --- | --- | --- | --- | --- | --- | --- | --- | --- | --- |
|  | OR | OR | 95% CI | P value ^a^ | OR | 95% CI | P value ^a^ | OR | 95% CI | P value ^a^ |
| All cardiovascular events ^b^ | | | | | | | | | | |
| Model 1 | 1.00 (reference) | 2.01 | 1.96-2.06 | <0.001 | 8.81 | 8.55-9.08 | <0.001 | 11.42 | 11.14-11.70 | <0.001 |
| Model 2 | 1.00 (reference) | 1.51 | 1.48-1.55 | <0.001 | 6.32 | 6.13-6.53 | <0.001 | 6.45 | 6.28-6.64 | <0.001 |
| Model 3 | 1.00 (reference) | 1.50 | 1.46-1.54 | <0.001 | 6.29 | 6.10-6.50 | <0.001 | 6.36 | 6.19-6.55 | <0.001 |
| Model 4 | 1.00 (reference) | 1.49 | 1.45-1.53 | <0.001 | 6.24 | 6.05-6.44 | <0.001 | 6.32 | 6.14-6.50 | <0.001 |
| Myocardial infarction | | | | | | | | | | |
| Model 1 | 1.00 (reference) | 1.81 | 1.76-1.87 | <0.001 | 7.42 | 7.19-7.66 | <0.001 | 8.78 | 8.55-9.02 | <0.001 |
| Model 2 | 1.00 (reference) | 1.30 | 1.26-1.34 | <0.001 | 4.91 | 4.75-5.08 | <0.001 | 4.40 | 4.27-4.53 | <0.001 |
| Model 3 | 1.00 (reference) | 1.28 | 1.24-1.32 | <0.001 | 4.88 | 4.72-5.05 | <0.001 | 4.32 | 4.19-4.46 | <0.001 |
| Model 4 | 1.00 (reference) | 1.28 | 1.24-1.32 | <0.001 | 4.84 | 4.68-5.00 | <0.001 | 4.29 | 4.16-4.42 | <0.001 |
| Stroke | | | | | | | | | | |
| Model 1 | 1.00 (reference) | 2.20 | 2.10-2.29 | <0.001 | 5.89 | 5.64-6.15 | <0.001 | 8.19 | 7.89-8.50 | <0.001 |
| Model 2 | 1.00 (reference) | 1.99 | 1.91-2.08 | <0.001 | 5.08 | 4.86-5.32 | <0.001 | 6.57 | 6.30-6.85 | <0.001 |
| Model 3 | 1.00 (reference) | 1.99 | 1.91-2.08 | <0.001 | 5.08 | 4.86-5.32 | <0.001 | 6.58 | 6.31-6.86 | <0.001 |
| Model 4 | 1.00 (reference) | 1.99 | 1.90-2.08 | <0.001 | 5.05 | 4.83-5.29 | <0.001 | 6.54 | 6.27-6.82 | <0.001 |

Abbreviations: CI, confidence interval; COPD, chronic obstructive pulmonary disease; HT, hypertension; OR: odds ratio; POAD, peripheral occlusive arterial disease.

^a^ The significance of OR for major cardiovascular events associated with constipation.

^b^ Cardiovascular events included myocardial infarction, angina, stroke and transient ischemic attack.

Model 1: Adjusted for age.

Model 2: Adjusted for age, and cardiovascular risk factors (obesity, smoking, diabetes, sleep apnoea, COPD, kidney disease, endocrine disorders, metabolic disorders, POAD, atrial fibrillation and cardiac arrhythmia).

Model 3: Adjusted for all the factors in Model 2 plus gastrointestinal disorders (irritable bowel syndrome, ulcerative colitis, Crohn’s disease and other gastrointestinal disorders).

Model 4: Adjusted for all the factors in Model 3 plus metropolitan residence.

**Supplementary Table 4**: Risk of cardiovascular events associated with constipation and hypertension in 295,522 female patients

|  | Without either condition (N=114,033) | With constipation alone  (N=74,237) | | | With hypertension alone  (N=33,728) | | | With both constipation and hypertension (N=73,524) | | |
| --- | --- | --- | --- | --- | --- | --- | --- | --- | --- | --- |
|  | OR | OR | 95% CI | P value ^a^ | OR | 95% CI | P value ^a^ | OR | 95% CI | P value ^a^ |
| All cardiovascular events ^b^ | | | | | | | | | | |
| Model 1 | 1.00 (reference) | 2.14 | 2.09-2.20 | <0.001 | 8.28 | 8.04-8.52 | <0.001 | 11.26 | 11.00-11.54 | <0.001 |
| Model 2 | 1.00 (reference) | 1.72 | 1.67-1.77 | <0.001 | 6.15 | 5.97-6.34 | <0.001 | 6.92 | 6.73-7.10 | <0.001 |
| Model 3 | 1.00 (reference) | 1.69 | 1.65-1.74 | <0.001 | 6.10 | 5.92-6.29 | <0.001 | 6.77 | 6.59-6.96 | <0.001 |
| Model 4 | 1.00 (reference) | 1.69 | 1.64-1.73 | <0.001 | 6.01 | 5.83-6.19 | <0.001 | 6.69 | 6.51-6.87 | <0.001 |
| Myocardial infarction | | | | | | | | | | |
| Model 1 | 1.00 (reference) | 1.96 | 1.90-2.03 | <0.001 | 7.48 | 7.23-7.74 | <0.001 | 9.52 | 9.25-9.81 | <0.001 |
| Model 2 | 1.00 (reference) | 1.49 | 1.43-1.54 | <0.001 | 4.98 | 4.80-5.16 | <0.001 | 5.01 | 4.85-5.17 | <0.001 |
| Model 3 | 1.00 (reference) | 1.46 | 1.41-1.51 | <0.001 | 4.93 | 4.75-5.11 | <0.001 | 4.88 | 4.72-5.04 | <0.001 |
| Model 4 | 1.00 (reference) | 1.45 | 1.40-1.50 | <0.001 | 4.85 | 4.68-5.03 | <0.001 | 4.82 | 4.67-4.98 | <0.001 |
| Stroke | | | | | | | | | | |
| Model 1 | 1.00 (reference) | 2.20 | 2.10-2.29 | <0.001 | 6.54 | 6.27-6.82 | <0.001 | 8.66 | 8.35-8.99 | <0.001 |
| Model 2 | 1.00 (reference) | 2.01 | 1.92-2.10 | <0.001 | 5.71 | 5.46-5.97 | <0.001 | 7.07 | 6.79-7.35 | <0.001 |
| Model 3 | 1.00 (reference) | 2.01 | 1.92-2.10 | <0.001 | 5.71 | 5.46-5.97 | <0.001 | 7.07 | 6.79-7.36 | <0.001 |
| Model 4 | 1.00 (reference) | 2.01 | 1.92-2.10 | <0.001 | 5.67 | 5.43-5.93 | <0.001 | 7.03 | 6.75-7.32 | <0.001 |

Abbreviations: CI, confidence interval; COPD, chronic obstructive pulmonary disease; HT, hypertension; OR: odds ratio; POAD, peripheral occlusive arterial disease.

^a^ The significance of OR for major cardiovascular events associated with constipation.

^b^ Cardiovascular events included myocardial infarction, angina, stroke and transient ischemic attack.

Model 1: Adjusted for age.

Model 2: Adjusted for age, and cardiovascular risk factors (obesity, smoking, diabetes, sleep apnoea, COPD, kidney disease, endocrine disorders, metabolic disorders, POAD, atrial fibrillation and cardiac arrhythmia).

Model 3: Adjusted for all the factors in Model 2 plus gastrointestinal disorders (irritable bowel syndrome, ulcerative colitis, Crohn’s disease and other gastrointestinal disorders).

Model 4: Adjusted for all the factors in Model 3 plus metropolitan residence.

**Supplementary Table 5**: Risk of cardiovascular events in 194,063 patients with primary hypertension.

|  | OR | 95% CI | P value^b^ |
| --- | --- | --- | --- |
| All cardiovascular events ^c^ | | | |
| Model 1 | 1.30 | 1.28-1.33 | <0.001 |
| Model 2 | 1.32 | 1.29-1.34 | <0.001 |
| Model 3 | 1.12 | 1.10-1.15 | <0.001 |
| Model 4 | 1.11 | 1.09-1.14 | <0.001 |
| Model 5 | 1.11 | 1.09-1.14 | <0.001 |
| Myocardial infarction | | | |
| Model 1 | 1.20 | 1.18-1.22 | <0.001 |
| Model 2 | 1.22 | 1.19-1.24 | <0.001 |
| Model 3 | 0.99 | 0.97-1.01 | 0.212 |
| Model 4 | 0.97 | 0.95-1.00 | 0.019 |
| Model 5 | 0.98 | 0.96-1.00 | 0.025 |
| Stroke | | | |
| Model 1 | 1.34 | 1.31-1.37 | <0.001 |
| Model 2 | 1.35 | 1.31-1.38 | <0.001 |
| Model 3 | 1.30 | 1.27-1.33 | <0.001 |
| Model 4 | 1.31 | 1.28-1.34 | <0.001 |
| Model 5 | 1.31 | 1.28-1.34 | <0.001 |

Abbreviations: CI, confidence interval; COPD, chronic obstructive pulmonary disease; HT, hypertension; OR: odds ratio; POAD, peripheral occlusive arterial disease.

^a^ Among 195,062 patients with hypertension, 592 had secondary hypertension and 407 had non-specified hypertension. This analysis only included 194,063 patients with primary hypertension which counted for 99.5% of patients with hypertension.

^b^ The significance of OR for major cardiovascular events associated with constipation.

^c^ Cardiovascular events included myocardial infarction, angina, stroke and transient ischemic attack.

Model 1: Adjusted for age.

Model 2: Adjusted for age and sex.

Model 3: Adjusted for age, sex, and cardiovascular risk factors (obesity, smoking, diabetes, sleep apnoea, COPD, kidney disease, endocrine disorders, metabolic disorders, POAD, atrial fibrillation and cardiac arrhythmia).

Model 4: Adjusted for all the factors in Model 3 plus gastrointestinal disorders (irritable bowel syndrome, ulcerative colitis, Crohn’s disease and other gastrointestinal disorders).

Model 5: Adjusted for all the factors in Model 4 plus metropolitan residence.

**Supplementary Table 6**: Risk of cardiovascular events associated with constipation and primary hypertension in 540,173 patients

|  | Without either condition (N=208,413) | With constipation alone (N=137,697) | | | With primary hypertension alone (N=61,785) | | | With both constipation and primary hypertension (N=132,278) | | |
| --- | --- | --- | --- | --- | --- | --- | --- | --- | --- | --- |
|  | OR | OR | 95% CI | P value ^a^ | OR | 95% CI | P value ^a^ | OR | 95% CI | P value ^a^ |
| All cardiovascular events ^b^ | | | | | | | | | | |
| Model 1 | 1.00 (reference) | 2.07 | 2.03-2.11 | <0.001 | 8.41 | 8.24-8.59 | <0.001 | 11.03 | 10.85-11.22 | <0.001 |
| Model 2 | 1.00 (reference) | 2.07 | 2.03-2.11 | <0.001 | 8.59 | 8.42-8.77 | <0.001 | 11.39 | 11.20-11.59 | <0.001 |
| Model 3 | 1.00 (reference) | 1.61 | 1.58-1.64 | <0.001 | 6.27 | 6.14-6.41 | <0.001 | 6.73 | 6.61-6.87 | <0.001 |
| Model 4 | 1.00 (reference) | 1.59 | 1.56-1.62 | <0.001 | 6.24 | 6.10-6.37 | <0.001 | 6.62 | 6.49-6.75 | <0.001 |
| Model 5 | 1.00 (reference) | 1.58 | 1.55-1.61 | <0.001 | 6.16 | 6.03-6.30 | <0.001 | 6.55 | 6.43-6.68 | <0.001 |
| Myocardial infarction | | | | | | | | | | |
| Model 1 | 1.00 (reference) | 1.87 | 1.83-1.91 | <0.001 | 7.31 | 7.14-7.48 | <0.001 | 8.83 | 8.66-9.01 | <0.001 |
| Model 2 | 1.00 (reference) | 1.87 | 1.83-1.91 | <0.001 | 7.47 | 7.30-7.64 | <0.001 | 9.15 | 8.98-9.33 | <0.001 |
| Model 3 | 1.00 (reference) | 1.37 | 1.34-1.41 | <0.001 | 4.95 | 4.83-5.07 | <0.001 | 4.69 | 4.59-4.80 | <0.001 |
| Model 4 | 1.00 (reference) | 1.35 | 1.32-1.38 | <0.001 | 4.91 | 4.79-5.03 | <0.001 | 4.59 | 4.49-4.70 | <0.001 |
| Model 5 | 1.00 (reference) | 1.35 | 1.31-1.38 | <0.001 | 4.85 | 4.74-4.98 | <0.001 | 4.55 | 4.45-4.66 | <0.001 |
| Stroke | | | | | | | | | | |
| Model 1 | 1.00 (reference) | 2.20 | 2.13-2.27 | <0.001 | 6.25 | 6.07-6.45 | <0.001 | 8.44 | 8.22-8.67 | <0.001 |
| Model 2 | 1.00 (reference) | 2.19 | 2.13-2.26 | <0.001 | 6.26 | 6.07-6.45 | <0.001 | 8.47 | 8.26-8.70 | <0.001 |
| Model 3 | 1.00 (reference) | 2.00 | 1.94-2.06 | <0.001 | 5.44 | 5.27-5.62 | <0.001 | 6.87 | 6.67-7.07 | <0.001 |
| Model 4 | 1.00 (reference) | 2.00 | 1.94-2.07 | <0.001 | 5.44 | 5.27-5.62 | <0.001 | 6.88 | 6.68-7.08 | <0.001 |
| Model 5 | 1.00 (reference) | 2.00 | 1.94-2.06 | <0.001 | 5.41 | 5.24-5.58 | <0.001 | 6.84 | 6.64-7.04 | <0.001 |

Abbreviations: CI, confidence interval; COPD, chronic obstructive pulmonary disease; HT, hypertension; OR: odds ratio; POAD, peripheral occlusive arterial disease.

^a^ The significance of OR for major cardiovascular events associated with constipation.

^b^ Cardiovascular events included myocardial infarction, angina, stroke and transient ischemic attack.

Model 1: Adjusted for age.

Model 2: Adjusted for age and sex.

Model 3: Adjusted for age, sex, and cardiovascular risk factors (obesity, smoking, diabetes, sleep apnoea, COPD, kidney disease, endocrine disorders, metabolic disorders, POAD, atrial fibrillation and cardiac arrhythmia).

Model 4: Adjusted for all the factors in Model 3 plus gastrointestinal disorders (irritable bowel syndrome, ulcerative colitis, Crohn’s disease and other gastrointestinal disorders).

Model 5: Adjusted for all the factors in Model 4 plus metropolitan residence.

**Supplementary Table 7**: ICD10 codes for relevant diseases and conditions

| Constipation | K59.0 |
| --- | --- |
| Myocardial infarction | I21, I22, I23, I24, I25 |
| Angina | I200 |
| Stroke | G46, I60, I61, I62, I63, I64, I65, I66, I67 |
| Transient ischaemic attack | G450-453, G458-459 |
| Hypertension | I10, I11, I12, I13, I15 |
| Obesity | E66, U781 |
| Diabetes | E10, E11, E13, E14 |
| Sleep apnoea | G473, G474, G478, G479 |
| Smoking | Z716, Z720, Z812, Z8643, T652 |
| Atrial fibrillation and arrhythmia | I48, I49 |
| Peripheral occlusive arterial disease | I702, |
| Chronic obstructive pulmonary disease | U832 |
| Kidney Disease | I12, I13, I150, I151, N17, N18, N19, Q61, U871 |
| Endocrine Disorders | E34, G735, I152 |
| Metabolic Disorders | E88, E89, G736, I431, M141, N163, U782 |
| Irritable bowel syndrome | K58 |
| Ulcerative colitis | K51, U842 |
| Crohn’s disease | K50, U841 |
| Other gastrointestinal Disorders | A213, A222, B462, K63, K9, M9836, P543, Q6475, T478, T479, |
| Hypertension – Primary | I10 |
| Hypertension – Secondary | I15 |
| Hypertension – other | I11, I12, I13 |

**Supplementary Table 8:** The association of each individual risk factor with cardiovascular events in 541,172 patients, analysed by simple binary logistic regression

|  | OR | 95% CI | P value ^a^ |
| --- | --- | --- | --- |
| All cardiovascular events ^b^ | | | |
| Constipation | 2.50 | 2.47-2.53 | <0.001 |
| Age | 1.01 | 1.00-1.01 | <0.001 |
| Male sex | 1.44 | 1.42-1.46 | <0.001 |
| Obesity | 2.14 | 2.09-2.19 | <0.001 |
| Smoke | 2.05 | 2.02-2.07 | <0.001 |
| Hypertension | 7.21 | 7.12-7.30 | <0.001 |
| Diabetes | 2.24 | 2.21-2.27 | <0.001 |
| Sleep apnoea | 2.04 | 1.98-2.10 | <0.001 |
| COPD | 2.24 | 2.19-2.29 | <0.001 |
| Kidney disease | 3.55 | 3.50-3.59 | <0.001 |
| Endocrine disorder | 1.74 | 1.28-2.36 | <0.001 |
| Metabolic disorder | 1.81 | 1.76-1.87 | <0.001 |
| AFA | 4.26 | 4.26-4.31 | <0.001 |
| POAD | 3.85 | 3.75-3.95 | <0.001 |
| Irritable bowel syndrome | 1.55 | 1.43-1.67 | <0.001 |
| Ulcerative colitis | 1.54 | 1.44-1.64 | <0.001 |
| Crohn’s disease | 1.52 | 1.38-1.67 | <0.001 |
| Other gastrointestinal disorders | 1.78 | 1.76-1.81 | <0.001 |
| Metropolitan residence | 0.78 | 0.77-0.78 | <0.001 |
| Myocardial infarction | | | |
| Constipation | 2.24 | 2.21-2.27 | <0.001 |
| Age | 0.99 | 0.99-1.00 | <0.001 |
| Male sex | 1.67 | 1.65-1.69 | <0.001 |
| Obesity | 2.37 | 2.32-2.43 | <0.001 |
| Smoke | 2.28 | 2.25-2.31 | <0.001 |
| Hypertension | 6.22 | 6.13-6.30 | <0.001 |
| Diabetes | 2.33 | 2.30-2.36 | <0.001 |
| Sleep apnoea | 2.27 | 2.20-2.34 | <0.001 |
| COPD | 2.37 | 2.31-2.42 | <0.001 |
| Kidney disease | 3.76 | 3.71-3.81 | <0.001 |
| Endocrine disorder | 1.38 | 0.98-1.94 | 0.067 |
| Metabolic disorder | 1.83 | 1.77-1.90 | <0.001 |
| AFA | 4.09 | 4.03-4.14 | <0.001 |
| POAD | 3.90 | 3.80-4.00 | <0.001 |
| Irritable bowel syndrome | 1.50 | 1.38-1.63 | <0.001 |
| Ulcerative colitis | 1.63 | 1.51-1.75 | <0.001 |
| Crohn’s disease | 1.54 | 1.39-1.71 | <0.001 |
| Other gastrointestinal disorders | 1.85 | 1.82-1.87 | <0.001 |
| Metropolitan residence | 0.79 | 0.78-0.80 | <0.001 |
| Stroke | | | |
| Constipation | 2.40 | 2.36-2.45 | <0.001 |
| Age | 1.01 | 1.01-1.01 | <0.001 |
| Male sex | 1.15 | 1.13-1.16 | <0.001 |
| Obesity | 1.37 | 1.33-1.42 | <0.001 |
| Smoke | 1.52 | 1.49-1.54 | <0.001 |
| Hypertension | 5.23 | 5.13-5.32 | <0.001 |
| Diabetes | 1.72 | 1.69-1.75 | <0.001 |
| Sleep apnoea | 1.33 | 1.28-1.39 | <0.001 |
| COPD | 1.47 | 1.42-1.51 | <0.001 |
| Kidney disease | 2.26 | 2.22-2.29 | <0.001 |
| Endocrine disorder | 1.44 | 0.95-2.19 | 0.089 |
| Metabolic disorder | 1.51 | 1.44-1.57 | <0.001 |
| AFA | 2.78 | 2.73-2.83 | <0.001 |
| POAD | 2.51 | 2.43-2.59 | <0.001 |
| Irritable bowel syndrome | 1.28 | 1.15-1.43 | <0.001 |
| Ulcerative colitis | 1.20 | 1.08-1.32 | <0.001 |
| Crohn’s disease | 1.29 | 1.13-1.48 | <0.001 |
| Other gastrointestinal disorders | 1.42 | 1.40-1.45 | <0.001 |
| Metropolitan residence | 0.85 | 0.84-0.86 | <0.001 |

Abbreviations: AFA, Atrial fibrillation and arrhythmia; CI, confidence interval; COPD, chronic obstructive pulmonary; OR, odds ratio; POAD, peripheral occlusive arterial disease.

^a^ The significance of OR for major cardiovascular events associated with each individual factor.

^b^ Cardiovascular events included myocardial infarction, angina, stroke and transient ischemic attack.
